# Supplementary material for: AMPK Amplifies IL2–STAT5 Signaling to Maintain Stability of Regulatory T Cells in Aged Mice
Source: Int J Mol Sci. 2022 Oct 16;23(20):12384. doi: 10.3390/ijms232012384 (PMC9604214; doi:10.3390/ijms232012384)
Supplement: Supplementary file 1 [file ijms-23-12384-s001.zip › Supplementary Figures.pdf]

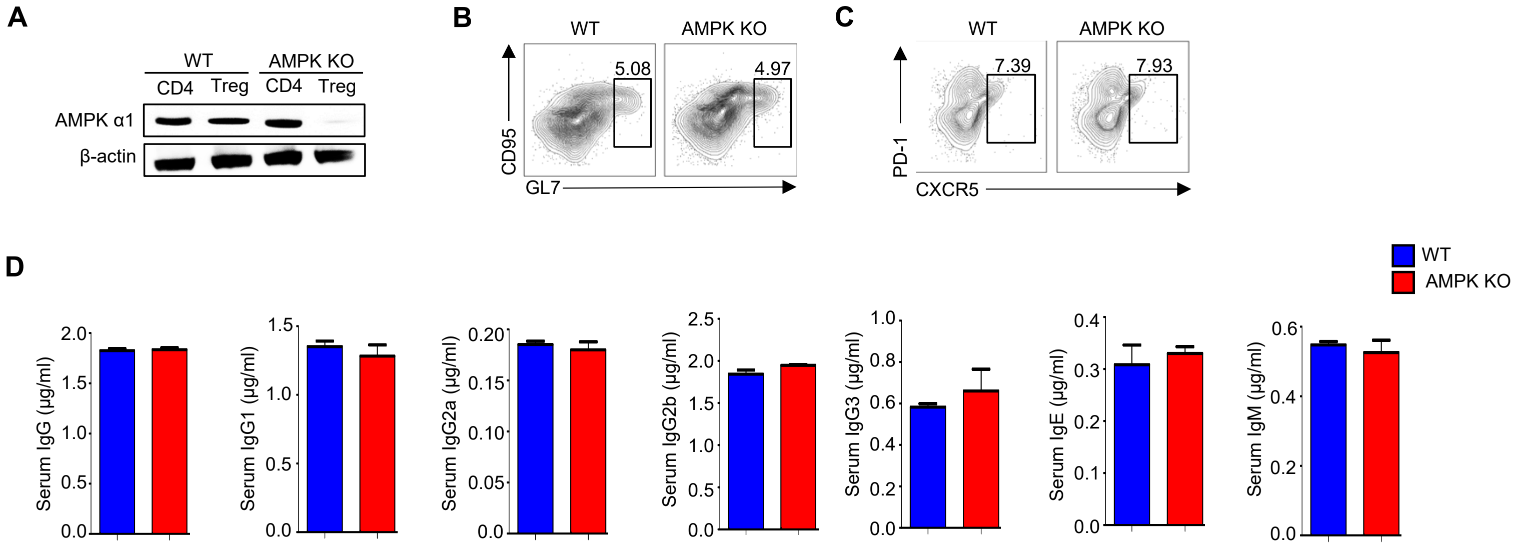

Figure S1: WT and AMPK Ko mice are comparable in terms of humoral immunity (A) Analysis of total AMPKα1 in CD4 and Tregs in WT and AMPK-KO mice by western blotting. **(B)** Representative flow cytometric plot showing the percentage of **(B)** germinal center B-cells and **(C)** Tfh cells in payer's patches of WT and AMPK-KO mice **(D)** Concentration of IgG (1:20000), IgG1 (1:20000), IgG2a (1:20000), IgG2b (1:20000), IgG3 (1:20000), IgE (1:20) and IgM (1:5000) in serum from WT and AMPK-KO mice.

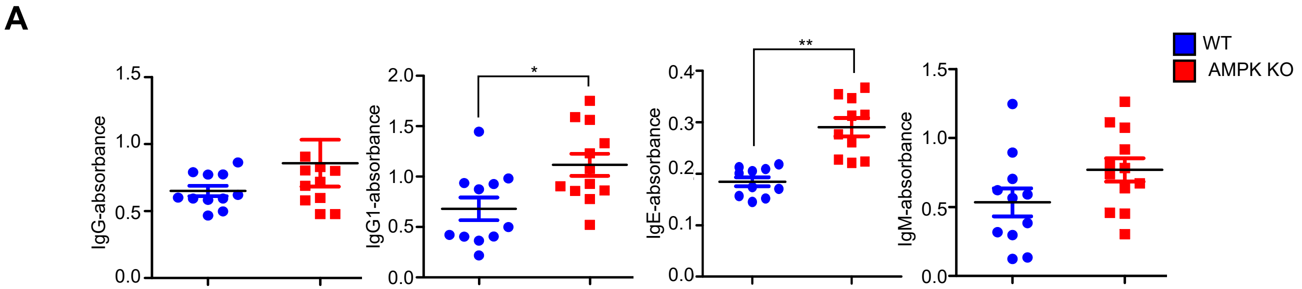

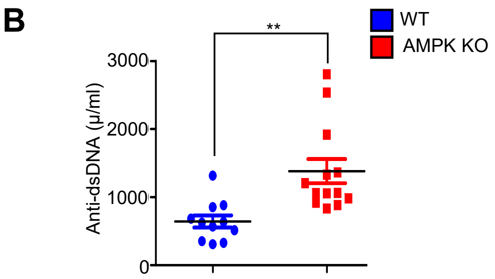

Figure S2: Alteration in humoral immunity in aged mice (A) Absorbance of IgG (1:20000), IgG1 (1:20000), IgG2a (1:20000), IgG2b (1:20000), IgG3 (1:20000), IgE (1:20) and IgM (1:5000) in serum from WT and AMPK-KO mice. **(B)** Concentration of antibodies to double stranded DNA in WT and AMPK-KO mice.

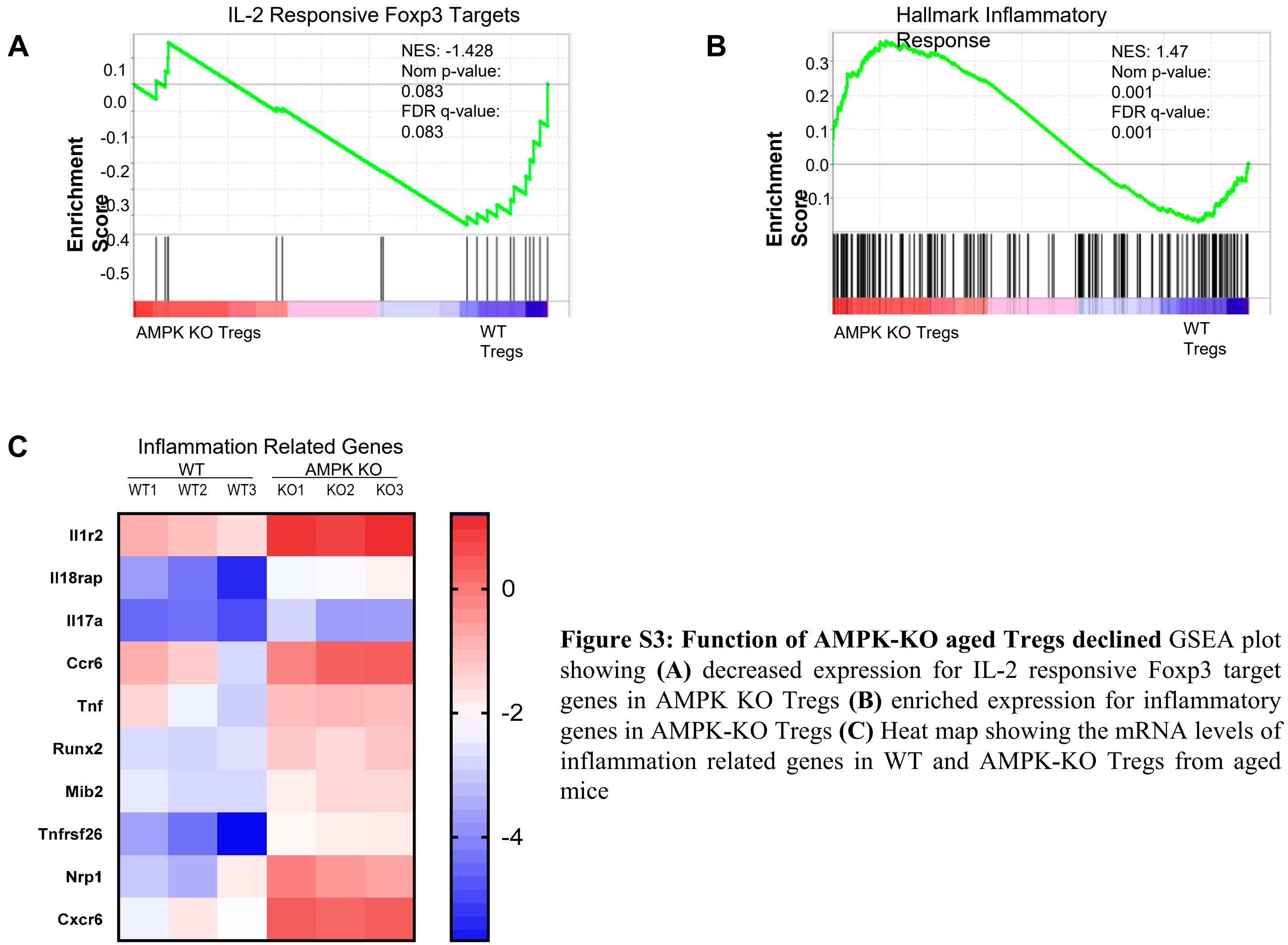

Figure S3: Function of AMPK-KO aged Tregs declined GSEA plot showing **(A)** decreased expression for IL-2 responsive Foxp3 target genes in AMPK KO Tregs **(B)** enriched expression for inflammatory genes in AMPK-KO Tregs **(C)** Heat map showing the mRNA levels of inflammation related genes in WT and AMPK-KO Tregs from aged mice
