# Supplementary material for: AMPK Amplifies IL2–STAT5 Signaling to Maintain Stability of Regulatory T Cells in Aged Mice
Source: Int J Mol Sci. 2022 Oct 16;23(20):12384. doi: 10.3390/ijms232012384 (PMC9604214; doi:10.3390/ijms232012384)
Supplement: Supplementary file 1 [file ijms-23-12384-s001.zip › Supplementary Table.pdf]

Table S1. The real-time PCR primer list, along with the sequences.

Species	Genes	Forward (5′-3′)	Reverse (5′-3′)
Mouse	Tbet	AGCAAGGACGGCGAATGTT	GGGTGGACATATAAGCGGTTC
Mouse	Rorγt	GGAGCTCTGCCAGAATGAGC	CAAGGCTCGAAACAGCTCCAC
Mouse	Foxp3	GGCCCTTCTCCAGGACAGA	GCTGATCATGGCTGGGTTGT
Mouse	Gata3	AGATGGCACGGGACACTACC	GTGGTGGTCTGACAGTTCGC
